# Lost to Follow-Up: Complications of an Invasive Giant Prolactinoma

**DOI:** 10.7759/cureus.9763

**Published:** 2020-08-15

**Authors:** Melodie M Blackmon, Andrea R Gilbert, John Floyd, Shaheryar Hafeez, Ali Seifi

**Affiliations:** 1 Department of Emergency Medicine, University of Texas Health Science Center at San Antonio, San Antonio, USA; 2 Department of Pathology, University of Texas Health Science Center at San Antonio, San Antonio, USA; 3 Department of Neurosurgery, University of Texas Health Science Center at San Antonio, San Antonio, USA

**Keywords:** infarction, pituitary adenoma, prolactinoma, proptosis, vasospasm

## Abstract

Invasive giant prolactinomas are a rare type of prolactin-secreting tumors. Most lactotroph adenomas, including giant prolactinomas, consist of the sparsely granulated subtype and respond well to medical therapy with dopamine agonists. Proptosis due to intra-orbital tumor extension and ischemic infarction are two rare complications associated with these tumors.

We report a case of a 51-year-old woman with a 30-year history of a macroprolactinoma who was lost to follow-up and returned with severe proptosis, a 10-cm invasive sellar mass on imaging, and markedly elevated serum prolactin levels, consistent with invasive giant prolactinoma. She was initially managed with dopamine agonists followed by palliative debulking of the tumor, which microscopically demonstrated a highly proliferative neoplasm predominantly consisting of sparsely granulated lactotroph adenoma with a minor component of the rare and aggressive acidophil stem cell adenoma subtype. Postoperatively, she developed a large left middle cerebral artery infarct and ultimately died.

This case is notable in that it demonstrates the aggressive nature of invasive giant prolactinomas when not treated and highlights two rare findings in patients with this tumor: orbital invasion and ischemic infarct.

## Introduction

Pituitary adenomas are benign tumors of the anterior pituitary, and prolactin-producing pituitary adenomas, so-called prolactinomas, are the most common type [1]. Microscopically, prolactinomas are classified into three subgroups: the common sparsely granulated lactotroph adenoma (SGLA), the rare densely granulated lactotroph adenoma (DGLA), and the exceptionally rare and poorly characterized acidophil stem cell adenoma (ASCA) [2]. Clinically, prolactinomas are categorized based on their size, with microadenomas measuring <1 cm, macroadenomas measuring >1 cm, and invasive giant prolactinomas, which are characterized by size >4 cm, prolactin >1,000 ng/mL, and symptoms of hyperprolactinemia or mass effect [1]. Invasive giant prolactinomas are rare, making up about 2%-3% of all prolactin-secreting tumors, and are most commonly seen in men, with a male to female ratio of 9:1.

Therapeutic goals for prolactinomas include normalization of hormone levels, reduction of tumor size, relief of mass effect, and restoration of gonadal function [1]. First-line treatment for prolactinomas, regardless of size, is medical therapy with dopamine agonists. Bromocriptine and cabergoline are most commonly used, and have shown to successfully reduce serum prolactin levels, restore gonadal function, reduce tumor size, and improve visual deficits in patients with prolactinomas, including those with invasive giant prolactinomas [3]. The improvement of visual deficits and headaches occurs for many patients within a matter of days. Due to having fewer side effects, a higher success rate, and a decreased likelihood of resistance, cabergoline is often the drug of choice. Surgical treatment, most often with transsphenoidal hypophysectomy, is another option if medical treatment fails or if the patient cannot tolerate the medications. Unfortunately, many patients who undergo surgical resection will have a recurrence of elevated prolactin levels. The overall long-term surgical cure rate is 61.1% with microprolactinomas and 26.2% with macroprolactinomas. Temozolomide and radiation have been used in aggressive tumors refractory to the aforementioned treatment modalities [3].

We present a case of a patient with an invasive giant prolactinoma who was lost to follow-up and presented with several unique features.

## Case presentation

The patient is a 51-year-old woman with a history of a prolactinoma that was diagnosed 30 years prior to her current admission when she presented to her primary care physician with a headache. She was lost to follow-up until five years prior to the current admission when she presented to a hospital with left eye ptosis and peripheral vision loss. Imaging at that time revealed a 3.6 cm × 3.3 cm × 3.2 cm mass in the sella turcica, and she underwent endoscopic endonasal excision of the pituitary adenoma. Postoperative CT scan after the initial operation showed a residual 2.7 cm × 3 cm × 2.6 cm sellar mass invading the left cavernous sinus. She was discharged from this hospitalization with instruction to take a dopamine agonist but was lost to follow-up and was not compliant with her medications for five years. Two months prior to the current admission, her condition was complicated by severe left eye proptosis with complete loss of vision and panhypopituitarism, and at this time she was started on cabergoline.

The patient now presented to the emergency department for intermittent episodes of staring associated with unresponsiveness. It was also reported that she had been increasingly lethargic and altered over the past month.

**Figure 1:**
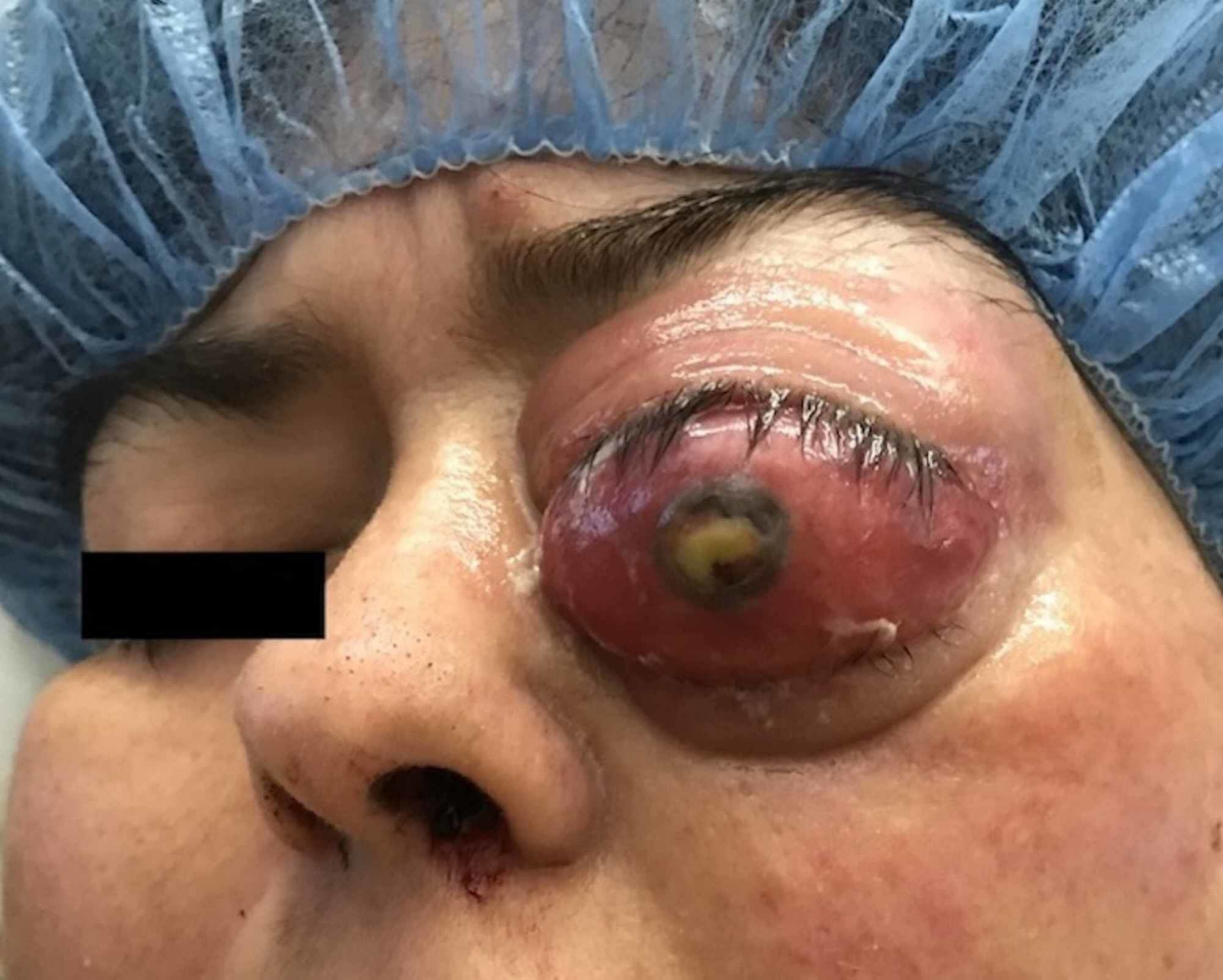
Severe proptosis and corneal ulceration of the left eye due to intra-orbital invasion of the prolactinoma.

On physical exam, the patient was morbidly obese with a body mass index of 45, somnolent, and oriented only to self. She had a right eye temporal visual field defect and severe left eye proptosis with erythema and corneal ulceration associated with complete visual loss (Figure 1). She had 4/5 strength to her left upper and lower extremity, but full strength in the other extremities, and was able to follow commands.

**Figure 2:**
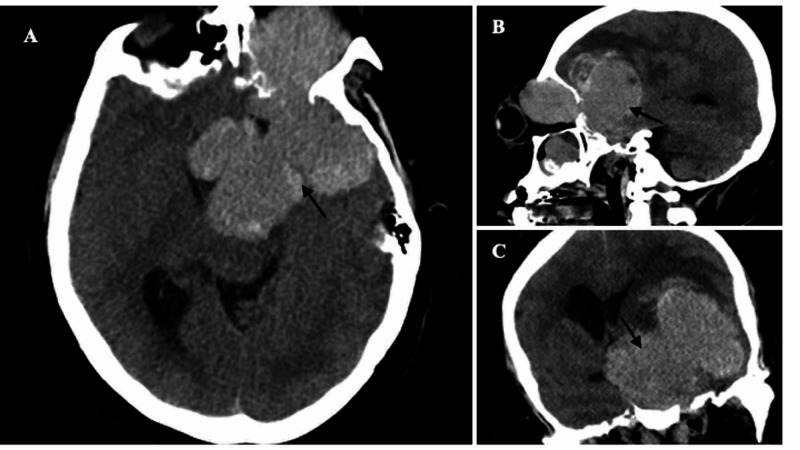
Non-contrast CT scan on presentation. (A) Axial, (B) sagittal, and (C) coronal views showing the 10 cm x 7 cm x 6 cm tumor invading the orbit, paranasal sinuses, and frontotemporal lobes and exerting mass effect on the brain parenchyma and brainstem.

CT scan of the head was done and showed a 10 cm × 7 cm × 6 cm sellar mass invading the left orbit, frontotemporal region, insular lobe, cavernous sinus, paranasal sinuses, and nasopharynx, with associated hemorrhagic necrosis, dilation of the right lateral ventricle, 1 cm of left to right midline shift, and mass effect on the brainstem (Figure 2). Her prolactin level was extremely high at 21,113 ng/mL. Thyroid-stimulating hormone was 0.7 microIU/mL, and free T4 was 0.7 ng/dL, consistent with central hypothyroidism. Lutenizing hormone was <0.01 mIU/mL, follicle-stimulating hormone 1.3 mIU/ml, and estradiol <19 pg/mL consistent with central hypogonadism. The patient was started on bromocriptine 10 mg daily, levothyroxine 175 mcg daily, hydrocortisone 20 mg in the morning and 10 mg at night, and levetiracetam 1,000 mg twice daily. 

Five days into her hospitalization, the patient became increasingly altered. Repeat CT scan of the head showed increasing hemorrhage and vasogenic edema, and worsening left to right midline shift. The patient was upgraded to the intensive care unit and was intubated due to her declining mental status and concern for inability to protect her airway. Medical management was continued, and four days later she underwent a left craniotomy for palliative debulking of the tumor. Neoplastic tissue was removed primarily from the retroorbital area to improve her proptosis, as well as to debulk some of the tumor burden in the frontal lobe.

**Figure 3:**
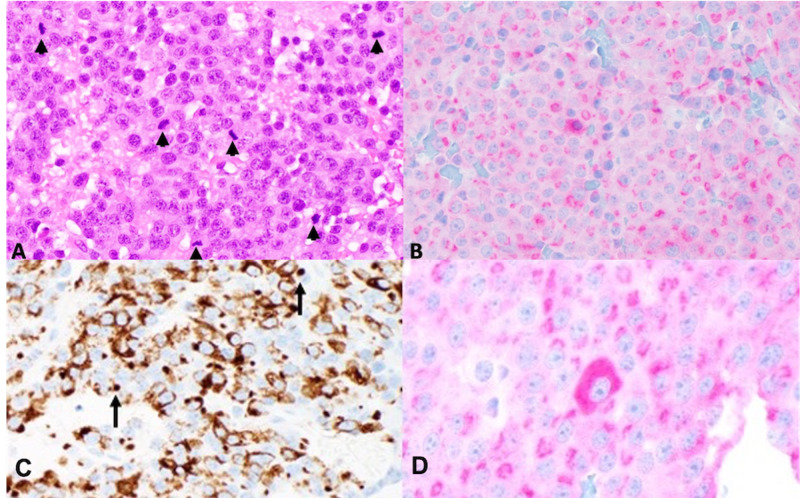
(A) Microscopic evaluation of the tumor revealed sheets of amphophilic cells with focal vacuolization and mitotic figures (black arrowheads) that were markedly increased in number (field diameter: 0.55 mm) (hematoxylin and eosin stain, ×400). (B) The majority of neoplastic cells show strong positive staining for prolactin in a Golgi pattern (prolactin IHC, ×400). (C) CAM5.2 stained most tumor cells in a perinuclear pattern, but also highlighted fibrous bodies (black arrows) (CAM5.2 IHC, ×400). (D) Prolactin also highlighted occasional, scattered tumor cells with enlarged cell bodies exhibiting diffuse cytoplasmic staining (prolactin IHC, ×400). IHC, immunohistochemistry

Histopathologic evaluation of the resected tissue with immunohistochemistry (IHC) revealed a synaptophysin-positive neuroendocrine neoplasm, and the majority of the neoplastic cells exhibited prolactin immunopositivity. These findings were suggestive of a SGLA mixed with another lactotroph adenoma subtype compatible with ASCA. Immunostains for other pituitary hormones were negative (Figure 3).

**Figure 4:**
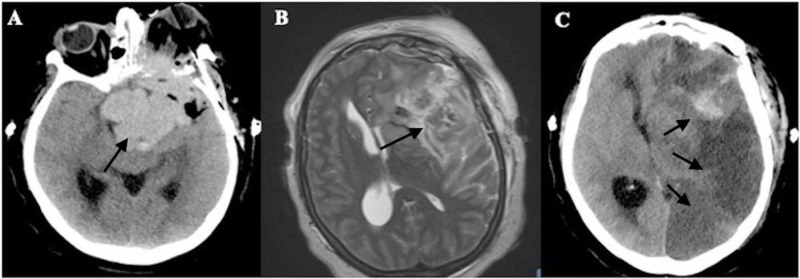
Postoperative imaging. (A) Non-contrast CT scan on postoperative day 0 showing decreased tumor size. (B) T2-weighted MRI scan on postoperative day 2. Axial view showing new area of infarction in the left middle cerebral artery (MCA) distribution. (C) Non-contrast CT scan on postoperative day 6 showing worsening left MCA infarction and left to right midline shift.

Immediately following the surgery, the patient was awake and alert, but she never regained her preoperative ability to follow commands. A postoperative CT scan showed a residual 7.9 cm × 6 cm × 4.4 cm tumor, as well as thin left frontal and parietal subdural and epidural hemorrhage, scattered surgical site pneumocephalus, and 1.4 cm of midline shift, but was otherwise unchanged from prior scans (Figure 4A). Two days postoperatively, she still had not regained the ability to follow commands, and was now decreasingly responsive, so MRI was done to evaluate for possible causes of this change in exam. MRI showed a new area of infarction in the left frontotemporal lobe, corresponding with the left middle cerebral artery (MCA) distribution, with associated severe cerebral edema and worsening midline shift, as well as encasement of multiple vascular structures including the left cavernous internal carotid arteries, bilateral anterior cerebral arteries, and left proximal MCA, and displacement of the bilateral posterior cerebral arteries (Figure 4B). Medical management was continued, but the patient’s exam continued to decline, and on postoperative day 6 she lost pupillary reflexes. Repeat CT scan showed progressing left MCA infarction with worsening vasogenic edema, 18 mm of left to right midline shift, and subfalcine herniation (Figure 4C). After discussion with her family, the patient was transitioned to hospice and died soon after extubation.

## Discussion

We present a case of a 51-year-old female with a macroprolactinoma who was lost to follow-up and returned many years later with an invasive giant prolactinoma complicated by invasion into the left orbit and a postoperative left MCA stroke. This case had several unique features, including intra-orbital extension with marked proptosis, postoperative ischemic stroke, and an intriguing histopathologic diagnosis. There are numerous reports in the literature of patients with invasive giant prolactinomas responding well to medical therapy, but to our knowledge, ours is one of the few to demonstrate an aggressive natural course in an untreated patient with a giant prolactinoma.

One of the major limitations of assessing the clinical behavior of prolactinomas as reported in the medical literature is that many authors do not subclassify these neoplasms. Lumping lactotroph subgroup under one heading nullifies the differences between them, which may offer an explanation for the wide range of reported clinical behavior. It is important to subclassify pituitary adenomas based on their histological features as some subtypes, such as the DGLA and ASCA, are known to exhibit aggressive behavior [3].

In this patient, the majority of the tumor was composed of SGLA, which is the most common prolactinoma subtype and typically shows a good response to medical management [3]. However, a minor component of the neoplasm consisted of morphologically distinct cells and fibrous bodies, compatible with ASCA. This finding suggests that, over the years in which the patient was lost to follow-up, a minor subset of the tumor may have undergone divergent differentiation into a more aggressive lactotroph adenoma subtype; to our knowledge, this has not been reported in the medical literature. 

The patient’s course also had unique clinical features. Orbital extension of prolactinomas is very rare; based on our review, there are only 14 other cases reported in the literature, with only 4 of them being in females [4-11]. In one reported case, the patient presented with isolated eye pain and proptosis in the absence of any other symptoms or evidence of hormone hypersecretion, making the diagnosis initially difficult [4]. It is important to recognize pituitary masses as a cause of proptosis and to consider this in the differential diagnosis of patients presenting with this finding. 

Ischemic stroke is another very rare complication of large pituitary tumors, and to our knowledge, there have only been 34 cases reported in the literature, with 4 occurring in the absence of apoplexy, such as in our case [12-14]. There are two proposed mechanisms for ischemic strokes in patients with pituitary tumors: mechanical compression and induced vasospasm [13]. Vasospasm is thought to occur secondary to blood in the subarachnoid space, vasoactive substances released from the tumor directly, or from direct vascular wall injury or hypothalamic injury during surgery.

In the cases noted above, the cerebral ischemia was the cause for the patient’s presentation to the hospital; it is noteworthy to address that the patient in our report did not have her stroke until after surgery. There are only 11 reported cases of ischemic infarction occurring after surgical removal of a pituitary mass, 10 of which occurred in the setting of vasospasm and 1 in the setting of thrombosis [15-18]. Vasospasm is a rarely documented complication after pituitary surgery, with 30 cases reported in the literature [19,20]. According to a literature review done by Alzhrani et al., the average onset of vasospasm was eight days after surgery [19]. This is consistent with findings described by Eseonu et al. who reported the average onset of vasospasm between eight and nine days, with a range of 3-14 days [20].

Prior to surgery, the patient was alert and following commands, but she never regained the ability to follow after surgery. Her immediate postoperative CT scan showed no evidence of cerebral infarction, but both MRI and CT on postoperative day 2 showed a large area of infarct. Based on the imaging and the timing of her change in exam, we propose that her stroke happened either during or soon after surgery. This timing of our patient’s stroke makes it highly unlikely that vasospasm was the cause of her ischemia. The patient’s invasive tumor encased many vascular structures, including the left MCA, and we propose that this, in addition to the increasing cytotoxic edema, produced mass effect on the MCA leading to her infarction.

We did not perform cerebral angiography or transcranial Doppler on our patient to further evaluate the cause of her stroke, as neither would have changed our management due to her advanced disease. It is important to note however that either of these can be done to definitively determine the cause of the ischemia; this is significant as treatment options vary based on the underlying cause. In one very small retrospective study done in patients with pituitary adenoma removal, all patients with a postoperative decline in Glasgow Coma Scale (GCS) were found to have either ischemic infarct or hemorrhage, both associated with vasospasm [17]. This highlights the importance of considering these as possibilities in patients with a postoperative decline in decline in mental status and obtaining the appropriate imaging in a timely manner to allow for intervention.

## Conclusions

To our knowledge, our case is one of the few to demonstrate the potentially aggressive nature of untreated invasive giant prolactinomas. Microscopically, the tumor showed a remarkable histopathology featuring predominantly SGLA subtype mixed with a minor population of prolactin-positive cells compatible with the rare and aggressive ASCA. The microscopic findings suggest that, over the years that the patient was lost to follow-up, the neoplasm may have de-differentiated from a benign lactotroph adenoma subtype to a more aggressive one; to our knowledge, this has not been reported in the literature.

This case is also remarkable for the severity and rarity of the sequelae that complicated this patient’s clinical course. Although proptosis is a rare complication of pituitary adenoma, neoplastic orbital invasion should be considered in the differential diagnosis of any patient with proptosis of unknown etiology. Another exceptional complication of invasive giant prolactinomas that can rarely occur after surgery is ischemic stroke, which should be considered in any patient with a postoperative decline in mental status or new focal neurologic deficits after surgery. The aggressive clinical course displayed by this neoplasm highlights the need for coordinated multidisciplinary management of such patients, whereas the microscopic findings highlight the need for histopathological subtyping of pituitary adenomas in order to better characterized their clinical behavior. These multifactorial issues require timely management with a multidisciplinary approach in order to optimize therapeutic outcomes for the patient. 
